# Host JDP2 expression in the bone marrow contributes to metastatic spread

**DOI:** 10.18632/oncotarget.5648

**Published:** 2015-10-14

**Authors:** Yelena Barbarov, Michael Timaner, Dror Alishekevitz, Tsonwin Hai, Kazunari K. Yokoyama, Yuval Shaked, Ami Aronheim

**Affiliations:** ^1^ Department of Cell Biology and Cancer Science, the B. Rappaport Faculty of Medicine, Technion-Israel Institute of Technology, Haifa, Israel; ^2^ Department of Biological Chemistry and Pharmacology, The Ohio State University, Columbus, Ohio, USA; ^3^ Graduate Institute of Medicine, Kaohsiung Medical University, Kaohsiung, Taiwan

**Keywords:** JDP2, metastasis, CCL5, bone marrow derived cells, lewis lung carcinoma

## Abstract

The c-Jun Dimerization Protein 2, JDP2, is a basic leucine zipper protein member of the activator protein-1 (AP-1) family of transcription factors. JDP2 typically suppresses gene transcription through multiple mechanisms and plays a dual role in multiple cellular processes, including cell differentiation and proliferation which is dependent on AP-1 function. Whereas the role of JDP2 expression within cancer cells has been studied, its role in stromal cells at the tumor microenvironment is largely unknown. Here we show that mice lacking JDP2 (JDP2−/−) display a reduced rate of metastasis in Lewis lung carcinoma (LLC) and polyoma middle T-antigen (PyMT) breast carcinoma mouse models. The replacement of wild-type bone marrow derived cells (BMDCs) with JDP2-deficient BMDCs recapitulates the metastatic phenotype of JDP2−/− tumor-bearing mice. *In vitro*, conditioned medium of wild-type BMDCs significantly potentiates the migration and invasion capacity of LLC cells as compared to that of JDP2−/− BMDCs. Furthermore, wild-type BMDCs secrete CCL5, a chemokine known to contribute to metastasis, to a greater extent than JDP2−/− BMDCs. The supplementation of CCL5 in JDP2−/− BMDC conditioned medium was sufficient to potentiate the invasion capacity of LLC. Overall, this study suggests that JDP2-expressing BMDCs within the tumor microenvironment contribute to metastatic spread.

## INTRODUCTION

Tumor growth is dependent on both the proliferation of cancer cells as well as the infiltration of different host cells which together with resident stromal cells constitute the tumor microenvironment. The interaction between cancer and stromal cells creates a permissive environment which promotes tumor growth and metastasis [1, 2]. The tumor stroma is composed of fibroblasts, endothelial cells, pericytes, and immune cells among others. The cross-talk between the different cell types in the tumor microenvironment determines cancer cell fate. For example, bone marrow derived cells (BMDCs) such as macrophages, dendritic cells, neutrophils, myeloid derived suppressor cells (MDSCs), T cells, B cells and natural killer cells (NKs) infiltrate the tumors and play crucial roles in cancer progression [3, 4]. Tumor associated macrophages (TAMs) secret growth factors that can promote angiogenesis, invasion, migration, metastatic spread and immunosuppression [5]. Likewise, tumor associated neutrophils (TANs) synthesize various cytokines and chemokines as well as pro-angiogenic factors [3]. Both macrophages and neutrophils are also sources of toxic substances capable of killing invading pathogens and cancer cells. The delicate balance between the different stromal cells within the tumor microenvironment has a dramatic impact on tumor growth and metastasis. Studying the role of the stroma, and especially BMDCs, in growing tumors can provide a greater understanding of tumor development and may lead to the development of novel treatment strategies. Here we focused on the expression of the c-Jun dimerization protein, 2 (JDP2) transcription repressor in the host and studied the effect of JDP2 ablation on tumor and metastatic growth.

JDP2 was isolated based on its ability to associate with c-Jun [6], reviewed in [7]. JDP2 encodes an 18 kDa bZIP protein that specifically interacts with c-Jun and ATF2 [6, 8]. JDP2 can bind TRE/CRE DNA elements as a homo- or hetero-dimer [6, 8]. Upon dimerization with c-Jun, DNA binding is potentiated, but transcription is inhibited [6]. JDP2 inhibits transcription by multiple mechanisms [6, 8–12]. Importantly, JDP2 can act as a transcriptional activator depending on the protein binding partner, specifically with the bZIP family member CHOP10 [13] or serve as co-activator when associates with members of the steroid hormone receptor family [14].

JDP2 is involved in cell differentiation processes, such as differentiation of skeletal muscle cells [15], adipocytes [16], and osteoclasts [17]. Potentiation of cell differentiation is facilitated by the ability of JDP2 to induce cell cycle withdrawal [15]. Various studies suggest that JDP2 has a dual role in malignant transformation. On the one hand, it is well established that JDP2 counteracts activator protein-1 (AP-1) transcription [6], and thus may interfere with the oncogenic properties of c-Jun. Indeed, JDP2 expression was found to inhibit cell transformation induced by Ras *in vitro* and in prostate cancer xenografts injected into SCID mice [18]. In addition, JDP2 suppresses cell cycle progression by down-regulation of cyclin-A2 [19]. On the other hand, JDP2 has been identified as a candidate oncogene in a high-throughput screen based on viral insertional mutagenesis in mice [20–22]. Consistently, tetracycline regulated transgenic mice expressing JDP2 in liver tissue exhibited higher mortality rate and increased number and size of tumors when compared with their wild-type counterparts in hepatocellular carcinoma mouse model [23]. Collectively, JDP2 expression within the cancer cells plays a dichotomous role in cancer progression.

Whereas much is known regarding JDP2 expression within cancer cells, the role of JDP2 in the stroma and how it affects cancer growth and metastasis is largely unknown. Here, we describe the role of JDP2 in host cells and its effects on tumorigenesis. We found that JDP2 expression in the host suppresses primary tumor growth; however, it promotes metastatic spread. These metastatic effects are partially mediated by BMDCs colonizing the primary tumor site and further secreting the pro-metastatic chemokine, CCL5.

## RESULTS

### Host-derived JDP2 expression promotes metastasis

**Figure 1:**
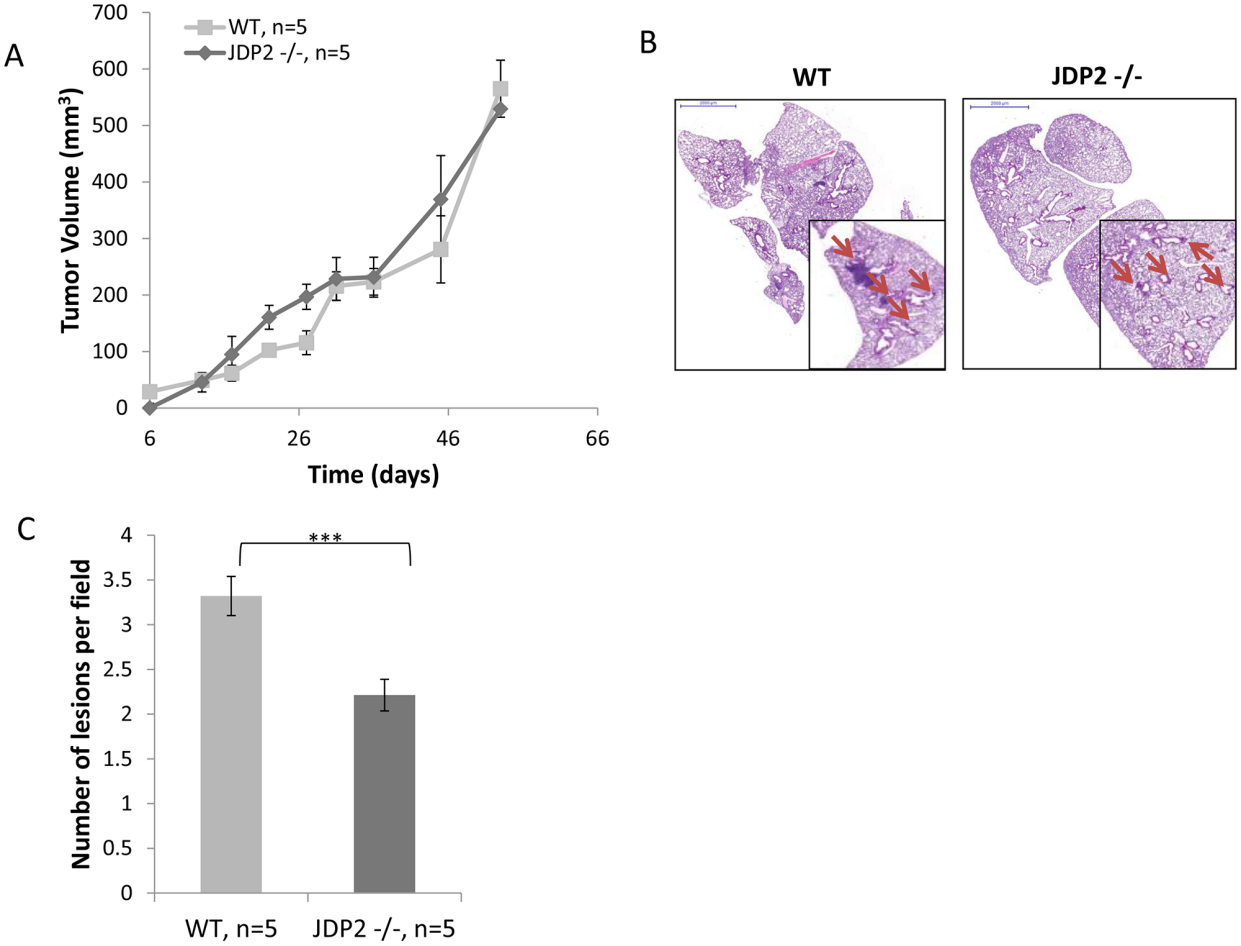
Host derived JDP2 expression promotes metastasis of mammary tumors **A.** Six-to-eight week old female WT and JDP2 −/− mice were orthotopically implanted to the mammary fat pad with 2 × 10^6^ PyMT cells mixed with Matrigel, and tumor volume was monitored over time. B-C. When tumors reached an average volume of 600 mm^3^, mice were sacrificed and lungs were harvested. Lungs were embedded in paraffin, sectioned, and subsequently stained with H&E. Arrows indicate metastatic lesions. Scale bars = 2000 μm. Small micrographs are 2X magnification. **B.** The number of pulmonary metastatic lesions per field was quantified (*n* > 6/group) C.***, *p* < 0.001 of a two-tailed *t*-test.

To characterize the impact of host JDP2 expression on metastasis, wild-type and JDP2 knockout mice (JDP2−/−) were orthotopically implanted into the mammary fat pads with polyoma middle T-antigen (PyMT) breast carcinoma cells. Tumor size was monitored over time and mice were sacrificed when the primary tumors reached an average size of 600 mm^3^. Wild-type and JDP2−/− mice developed primary tumors at a similar rate (Figure 1A). However, the number of metastatic lesions in the lungs of wild-type mice was significantly higher than that in JDP2−/− mice (Figure 1B–1C).

**Figure 2:**
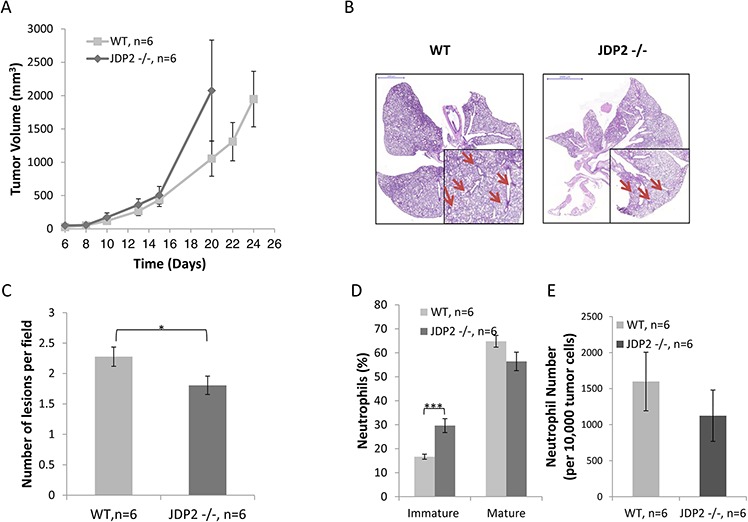
Host derived JDP2 expression inhibits tumor growth but promotes metastasis **A.** Six-to-eight week old wild-type (WT) and JDP2 −/− mice were subcutaneously implanted with 0.5 × 10^6^ LLC cells, and tumor volume was monitored over time. **B–E.** When tumors reached an average volume of 2000 mm^3^, mice were sacrificed and tumors and lungs were harvested. Lungs were embedded in paraffin, sectioned, and subsequently stained with H&E. Arrows indicate metastatic lesions Scale bars = 2000 μm. Small micrographs are 2X magnification. (B) The number of pulmonary metastatic lesions per field was quantified (C) Tumors were prepared as single cell suspensions and the percentage of mature (CD11b^+^Ly6C^(low)^Ly6G^+^) and immature (CD11b^+^Ly6C^(high)^Ly6G^+^) neutrophils (D), as well as the number of total neutrophils count per 10,000 cells (E), were determined by flow cytometry. *, *p* < 0.05; ***p < 0.001 of a two-tailed *t* test.

In an additional model, in which Lewis lung carcinoma (LLC) cells were subcutaneously implanted into the flanks of wild-type and JDP2−/− mice, tumors from wild-type mice were found to be relatively smaller than tumors from JDP2 −/− mice, although these differences were not statistically significant (Figure 2A). In a parallel experiment, when size-matched tumors were removed, the number of metastatic lesions in the lungs of wild-type mice was significantly higher than that in JDP2−/− mice (Figure 2B–2C), similar to the PyMT breast cancer model. Taken together these results indicate that JDP2 expression in the host may account for an increased number of metastasis.

### Metastasis is inhibited in mice harboring JDP2-deficient bone marrow cells

Recent studies have indicated that inflammatory cells as well as other accessory cells in the tumor sites contribute to metastasis spread [3, 4]. We therefore assessed the colonization of BMDCs in LLC tumors grown in wild-type or JDP2−/− mice. The excised size-matched tumors (similar to Figure 2) were prepared as single cell suspensions and the presence of various inflammatory cells was assessed using flow cytometry. No significant differences were found in the percentage of T cells and macrophages in tumors derived from wild-type and JDP2−/− mice (Supplementary Figure S1). However, a significant increase was observed in the percentage of immature neutrophils, and a decrease was seen in the percentage of mature neutrophils in the tumors from JDP2−/− mice, when compared to tumors from wild-type mice (Figure 2D). The total number of neutrophils in tumors from both groups did not significantly change (Figure 2E). These results are consistent with the role of JDP2 in neutrophils maturation [24].

**Figure 3:**
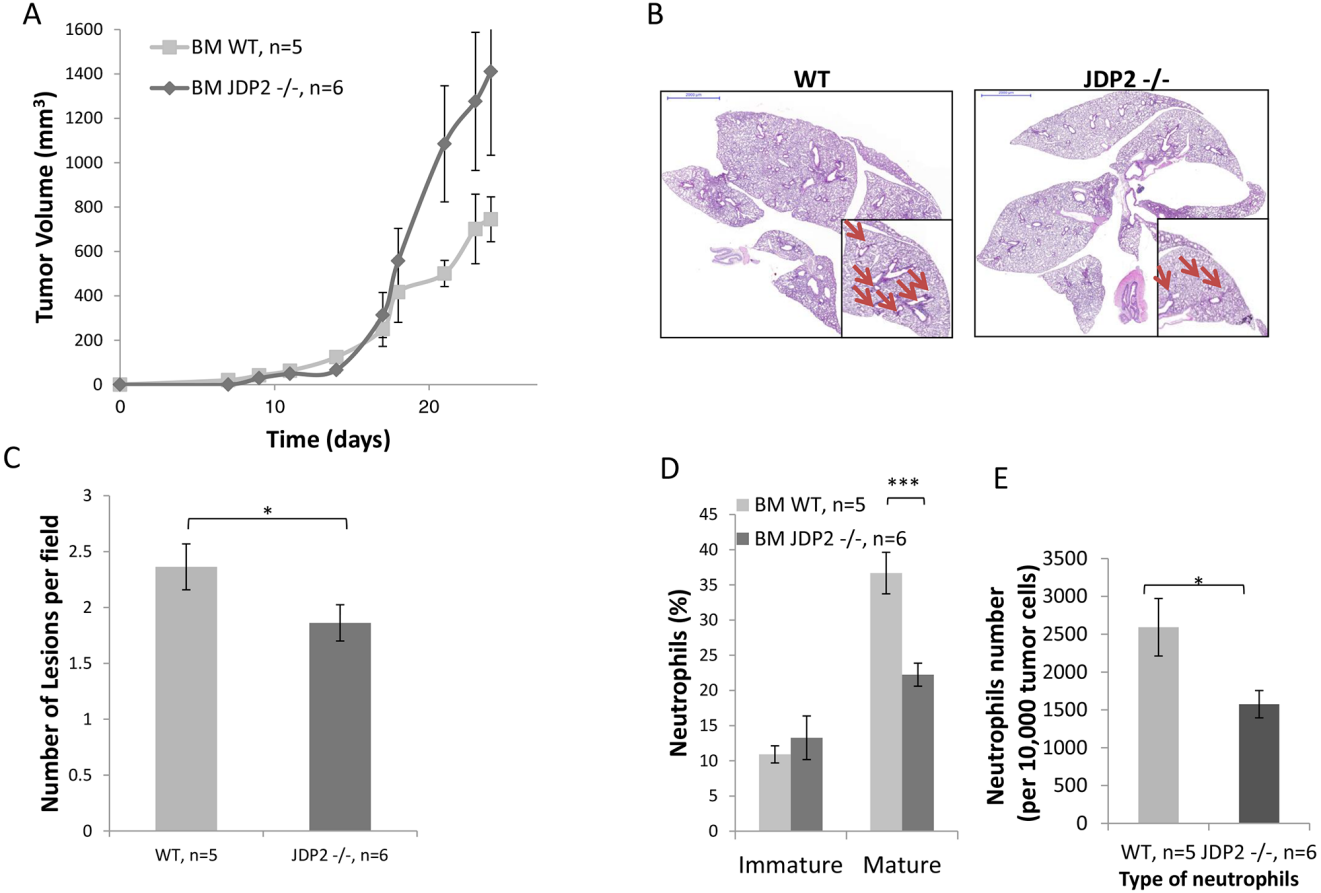
LLC-bearing mice harboring JDP2-deficient bone marrow exhibit reduced metastasis **A.** Wild-type C57B1/6 mice were transplanted with BMDCs from either wild-type (WT) or JDP2−/− mice. The chimeric mice were then subcutaneously implanted with 0.5 × 10^6^ LLC cells, and tumor volume was monitored over time. **B–E.** At end point, tumors and lungs were excised. Lungs were embedded in paraffin, sectioned, and subsequently stained with H&E. Arrows indicate metastatic lesions. Scale bars = 2000 μm. Small micrographs are 2X magnification (B) The number of pulmonary metastatic lesions per field was quantified (C) Tumors were prepared as single cell suspensions and the percentage of mature (CD11b^+^Ly6C^(low)^Ly6G^+^) and immature (CD11b^+^Ly6C^(high)^Ly6G^+^) neutrophils (D), as well as the total number of neutrophils per 10,000 cells (E), were determined by flow cytometry. *, *p* < 0.05; ***p < 0.001 of a two-tailed *t*-test.

Next, we performed a bone marrow transplantation experiment in which lethally irradiated wild-type mice were transplanted with BMDCs from JDP2−/− or wild-type mice. The efficiency of bone marrow transplantation was validated following bone marrow reconstitution (approximately 6–8 weeks) (data not shown). Subsequently, LLC cells were then subcutaneously implanted into the flanks of the chimeric mice and tumor growth was assessed. Chimeric mice transplanted with JDP2−/− bone marrow exhibited increased LLC tumor growth in comparison to control mice transplanted with wild- type bone marrow (Figure 3A). These findings are in agreement with the results shown in Figure 2A. Consistently, the number of metastatic lesions in chimeric mice harboring JDP2−/− BMDCs was significantly lower than that in the wild-type counterparts (Figure 3B–3C). Moreover, flow cytometry analysis of cells from tumors prepared as single cell suspensions revealed a significantly lower level of mature neutrophils and a decrease in neutrophils count in mice harboring JDP2−/− BMDCs than in control mice harboring wild-type BMDCs (Figure 3D–3E). Collectively, these results suggest that the expression of JDP2 in BMDCs account for the difference metastatic phenotype between wild-type and JDP2−/− host.

### BMDCs from wild-type mice colonize tumors in higher numbers than BMDCs from JDP2−/− mice

**Figure 4:**
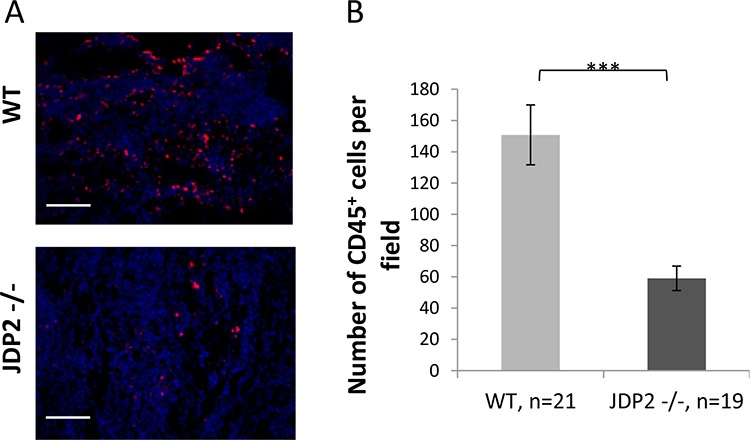
Wild-type BMDCs infiltrate into LLC tumors at a higher rate as compared with JDP2 −/− BMDCs **A.** OCT fixed LLC tumor slices were stained for αCD45, a pan hematopoietic marker (red). Nuclei were stained by DAPI (blue). Scale bar = 200 μm **B.** The number of CD45+BMDCs per filed was counted. ***p < 0.001 of a two-tailed *t*-test.

To further assess the contribution of wild-type and JDP2−/− BMDCs to tumor metastatic phenotype, we examined the potential recruitment of BMDCs to LLC tumors in the mouse model shown in Figure 2A. To this end, LLC tumors were stained with the BMDC pan-hematopoietic surface marker–CD45. LLC tumors in wild-type mice exhibited a substantial increase in CD45+ BMDC infiltration than tumors from JDP2−/− mice, suggesting that BMDCs are involved in the metastatic phenotype found in wild-type mice (Figure 4A–4B).

### JDP2 expressed in BMDCs is necessary to enhance cancer cell migration and invasion

**Figure 5:**
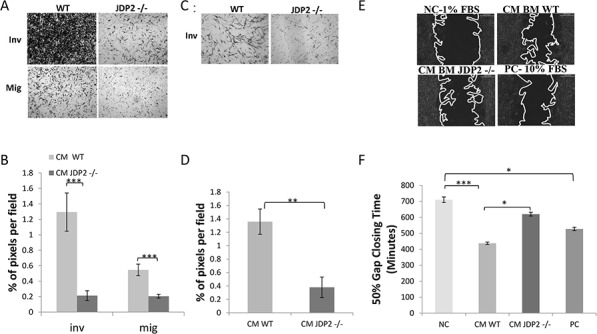
Conditioned medium of JDP2-expressing BMDCs promotes invasion and migration **A–B.** BMDCs of wild-type and JDP2−/− mice were cultured for 24 h to generate conditioned medium (CM). The migration and invasion properties of LLC cells were assessed in the presence of CM using the Boyden chamber assay. Cells invading (Inv) or migrating (Mig) through the membrane were stained with crystal violet and images were captured (A). The percentage of cell coverage per field was quantified (*n* > 8 fields/group) (B). **C–D.** The invasion properties of PyMT cells were assessed in the presence of CM derived from wild-type and JDP2−/− BMDC cultures by the modified Boyden chamber assay, as described in (A–B) **E–F.** PyMT cell motility was assessed by migration closure assay in the presence of CM derived from wild-type and JDP2−/− BMDC cultures. Serum-free and serum-rich medium were used as negative and positive controls (NC and PC), respectively. Gap closure was monitored over time. Representative images are shown when 50% of the gap was closed (E). The time necessary to achieve 50% gap closure is shown (*n* > 11/group) (F). *p < 0.05; **, *p* < 0.01; ***p < 0.001 of a one way ANOVA followed by Tukey post-hoc test when a comparison between more than two groups was performed, and unpaired student *t*-test when comparison between two groups was performed.

One possible mechanism by which JDP2 expression in BMDCs contributes to metastasis is by regulating the secretion of soluble factors that affect cancer cell metastatic properties. To test this possibility, we examined cancer cell migration and invasion in the presence of conditioned medium (CM) from wild-type and JDP2−/− BMDCs using the Boyden chamber assay. As shown in Figure 5A–5B, wild-type BMDC CM increased both invasion and migration of LLCs when compared to JDP2−/− BMDC CM. Similarly, the wild-type BMDC CM is more efficient than the JDP2−/− BMDC CM in promoting PyMT cell invasion but not migration (Figure 5C–5D, and data not shown). We also used the scratch wound assay in order to examine PyMT cell motility. Time-lapse analyses showed a faster wound closure in the presence of wild-type BMDC CM than in JDP2−/− BMDC CM (Figure 5E–5F). These results suggest that soluble factor(s) secreted by JDP2-expressing BMDCs promotes invasion and migration of cancer cells.

MMP9 is known to contribute to metastasis, and can be secreted by both cancer cells and BMDCs [25]. We therefore analyzed the ability of LLC cells to secrete MMP9 upon exposure to CM from wild-type or JDP2−/− BMDCs. We exposed LLC cells to BMDC CM for 24 hours, replaced the CM with serum free (SF) media and examined MMP9 activity in the SF media 24 hours later. Supplementary Figure S2 shows that pre-exposure to wild-type or JDP2−/− BMDC CM made no difference in MMP9 activity. We then examined the ability of wild-type or JDP2−/− BMDCs to secrete MMP9, and yet again we found no significant difference between the two groups (Supplementary Figure S3). Overall, these results indicate that MMP9 does not contribute to the metastatic phenotype induced by JDP2-expressing BMDCs.

### CCL5 is highly expressed in conditioned medium of wild-type BMDCs

**Figure 6:**
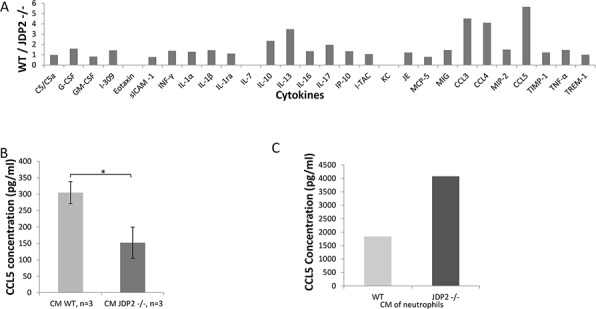
CCL5 is secreted by the JDP2-expressing BMDCs and is functionally important **A.** A mini cytokine array was probed with conditioned medium derived from wild-type and JDP2 −/− BMDCs. The level of each cytokine was assessed by densitometry. Shown is the ratio of wild-type to JDP2−/− groups. B-C. The level of CCL5 in conditioned medium derived from wild-type and JDP2−/− BMDCs (*n* = 3 mice/group) **B.** or conditioned medium derived from isolated neutrophils (pooled sample from 6 mice/group) **C.** was determined by ELISA. *p < 0.05 assessed by two-tailed *t*-test.

Next, we performed a candidate search to identify secreted factors that may account for the difference between wild-type and JDP2−/− BMDCs. A cytokine array, containing antibodies against 36 cytokines and chemokines, was probed with CM of wild-type or JDP2−/− BMDCs. Most of the factors exhibited similar signal intensity between the two groups. However, the signals for RANTES (CCL5), MIP1α (CCL3) and MIP1β (CCL4) were 4–5-fold higher in wild-type BMDC CM than in JDP2−/− BMDC CM (Figure 6A). The array data was validated with ELISA specific for CCL3, CCL4, and CCL5. The results in Figure 6B and Supplementary Figure S4 show that the level of CCL5 was two-fold higher in wild-type BMDC CM than in JDP2−/− BMDC CM, but no differences were observed in the levels of CCL3 and CCL4 between the two groups. Importantly, when focusing on CCL5, as it is known to promote breast cancer metastasis [26], we found that CCL5 was significantly increased in the CM of isolated neutrophils from JDP2−/− mice when compared to wild-type mice, as assessed by ELISA using pooled samples from 6 mice per group (Figure 6C).

We next asked whether the higher levels of CCL5 in JDP2 −/− neutrophils are related to the JDP2 direct repression of CCL5 transcription. We used a luciferase reporter assay, in which the luciferase gene transcription was placed under the control of the CCL5 regulatory region (−1036-+52) [27]. JDP2 was found to strongly repress luciferase reporter activity in the presence of CCL5 regulatory region (Supplementary Figure S5). These results suggest that the expression of CCL5 in neutrophils cannot explain the metastatic properties of tumor cells.

### CCL5 expressed by BMDCs is necessary and sufficient for LLC cell migration

**Figure 7:**
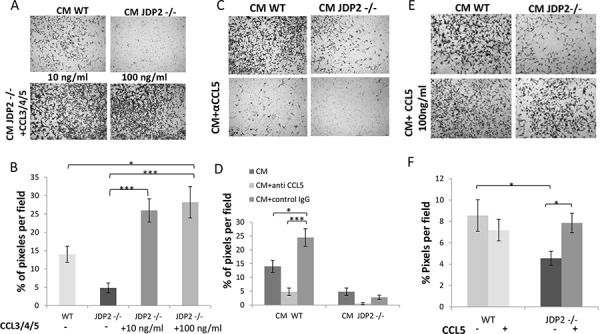
CCL5 is necessary and sufficient for increased LLC cell invasion **A.** The invasion properties of LLC cells were assessed by the Boyden chamber assay in the presence of JDP2 −/− BMDC CM supplemented with a mixture of purified CCL3, 4 and 5 (10 ng/ml and 100 ng/ml). **C.** CM from wild type or JDP2 −/− BMDCs in the presence of anti-CCL5 neutralizing antibodies or anti-cFos antibodies (as control) or E. CCL5 (100 ng/ml) added to the BMDC CM of either wild type or JDP2 −/− mice. The percentage of cell coverage per field was quantified (*n* > 8 fields/group) (**B, D** and **F,** respectively). *, *p* < 0.05; ***, *p* < 0.001 using one way ANOVA followed by a Tukey post-hoc test.

For cancer cells to respond to CCL5, they should express its receptor (CCR5). Thus, we examined CCR5 expression and found that it is expressed in LLC and PyMT cells (Supplementary Figure S6). To further assess the contribution of CCL5 expressed by BMDCs on metastatic phenotype, we asked whether CCL5 expressed by BMDCs is sufficient or necessary for the invasive properties of cancer cells. For this purpose, we tested LLC invasion in the presence of CM of BMDCs from JDP2−/− supplemented with CCL3, 4, 5. The addition of CCL3, 4, 5 to JDP2−/− BMDC CM resulted in increased LLC migration at a level which was even beyond wild-type BMDC CM (Figure 7A–7B).

Next, to assess the role of CCL5 on cancer cell invasion, we added neutralizing CCL5 antibodies or a control antibody into CM derived from wild-type and JDP2 −/− BMDCs. The CCL5 neutralizing antibodies but not control antibodies suppressed LLC invasion in both BMDC CM (Figure 7C–7D), indicating that CCL5 is necessary for tumor cell invasion. In a complementary experiment, the addition of CCL5 alone was sufficient for rescuing the invasion properties of tumor cells in the presence of JDP2−/− BMDC CM to a level comparable to that found in the wild-type BMDC CM (Figure 7E–7F). Collectively, these results indicate that CCL5 in JDP2 expressing BMDCs is necessary and sufficient for tumor cell invasion.

## DISCUSSION

Tumorigenesis requires a close interplay between cancer and host cells at the tumor microenvironment. Cancer cells are highly dependent on a constant supply of nutrients as well as growth factors and cytokines. The intimate cross-talk between cancer cells and immune and non-immune host cell types within the tumor microenvironment-collectively known as part of the tumor stroma-has a major effect on cancer progression and metastasis [28]. Various genes specifically expressed in BMDCs have been associated with tumor aggressiveness such as Ets2, Vegf, Hif1α, and Atf-3 [29–32]. Here, using mice harboring a homozygous mutation in the JDP2 allele, we characterized the role of JDP2 expression in non-cancer cells on cancer development and metastasis.

JDP2 is a transcription repressor protein which plays a role in maintaining cell homeostasis [7]. JDP2 is ubiquitously expressed in all cells tested and mediates the differentiation of numerous cell types [6, 7, 10]. Here we show that mice lacking JDP2 display a reduced rate of metastasis in lung and breast cancer models (LLC and PyMT, respectively). In the lung cancer model, we also observed the potentiation of tumor growth in mice lacking JDP2. The replacement of wild-type BMDCs with BMDCs from JDP2−/− mice in the LLC model recapitulated the phenotype, namely, higher tumor growth but lower numbers of metastatic lesions. We further observed that wild-type BMDCs infiltrated tumors in larger numbers than JDP2−/− BMDCs, and that the CM from JDP2−/− BMDCs had a reduced ability to promote LLC cell migration and invasion than the CM of wild-type BMDCs. Taken together, these results suggest that JDP2 expression within the host BMDCs plays a determinant role in cancer progression and metastasis.

We used a cytokine-chemokine antibody array to screen for differentially expressed soluble secreted factors from wild-type and JDP2−/− BMDCs. Higher levels of CCL 3, 4 and 5 were identified in CM derived from wild-type BMDCs when compared to JDP2−/− BMDCs. Significantly, the addition of CCL5 to CM of JDP2−/− BMDCs strongly potentiates the migration of LLC cells. Importantly, while we observed a decreased number of mature neutrophils in JDP2−/− mice, in accordance with previous publication [24], CCL5 levels were higher in neutrophils from JDP2−/− than in wild-type mice. This suggests that BMDC types, rather than neutrophils, may account for the metastatic phenotype observed. In addition, we found that JDP2 represses CCL5 promoter, and it is doing so, most likely through a CRE/AP-1 binding site located within the CCL5 promoter [27]. Interestingly, CCL5 was found to be a target gene for ATF3 [33], which is the most closely related basic leucine zipper protein and is highly homologous to JDP2 function [12].

CCL 3, 4 and 5 associate with the CCR5 G-protein coupled receptor, which is implicated in HER-2 positive breast cancer metastases [34]. CCR5 serves as a co-receptor for the membrane protein gp120 of the human immunodeficiency virus (HIV), and plays an important role for HIV entry of T cells. These findings led to the development of CCR5 antagonists. In cancer, CCR5 is expressed in a wide range of cancer cells, while its ligand is secreted by the host immune cells. CCL5-CCR5 axis may play a role in metastasis in a wide range of cancer cells and therefore may represent a novel therapeutic target [34]. Indeed, CCR5 antagonists were shown to block breast cancer cell invasion *in vitro* and lung metastasis in a mouse model of breast cancer [26]. In our study, we show that CCR5 is expressed by LLC and PyMT cells and that a CCL5 neutralizing antibody inhibits LLC cell invasion *in vitro*. Whether the neutralization of CCL5 or the blockade of CCR5 *in vivo* would inhibit metastasis in our system needs further investigation. Taken together, our study reveals a new role for JDP2 in the tumor stroma. Mainly, BMDCs expressing JDP2 home in large numbers to the tumor site and contribute to the secretion of the pro-metastatic CCL5 cytokine, leading to metastasis.

## MATERIALS AND METHODS

### Antibodies and nuclear staining

The following antibodies were purchased from Biolegend Inc.: CD11b (#50993), Ly6C (#128014), CD3ε (#100311), F4/80(#123110) and B220 (#103243). Ly6G (#551460), and CD45 (#553081) were purchased from BD Bioscience. αCCR5 (#NB110–55676) antibody used for Western blot analysis was purchased from Novus Biologicals. αGAPDH (#sc-25778) was obtained from Santa Cruz biotechnology Inc. Secondary Antibody, αRabbit, used for Western blot analysis was purchased from Sigma-Aldrich (#A0545). DAPI was used for nuclear staining (#0100–01, Southern Biotech). αCCL5 neutralizing antibody was purchased from Peprotech Inc. Rocky Hill, NJ.

### Cell lines

The Lewis lung carcinoma (LLC), HEK-293 and HT-1080 cell lines were purchased from the American Type Culture Collection (ATCC), and was used within 6 months of cell resuscitation. The PyMT breast cancer cell line was derived from primary tumor bearing transgenic mice expressing polyoma middle T-antigen (PyMT) under the control of the murine mammary tumor virus (MMTV) promoter [32]. LLC, PyMT, HT-1080 and human embryonic kidney 293 (HEK-293) cells were cultured in Dulbecco's Modified Eagle's Medium (DMEM) supplemented with 5% or 10%, respectively, (v/v) FBS, 1% streptomycin and penicillin, 1% L-glutamine and 1% sodium pyruvate at 37°C in a humidified atmosphere containing 5% CO_2_.

### Animal models

This study was carried out in strict accordance with the Guide for the Care and Use of Laboratory Animals of the National Institutes of Health. The protocol was approved by the Committee on the Ethics of Animal Experiments of the Technion (Permit Number: IL-074–06-2013). Surgeries were performed under sodium pentobarbital anesthesia, and all efforts were made to minimize suffering. The animals were fed standard rat chow containing 0.5% NaCl and tap water ad libitum. Wild-type C57B1/6 and JDP2−/− (JDP2 knock-out) mice [19] of the same genetic background were used in the study.

### Tumor implantation

LLC and PyMT cultured cells were harvested by trypsin treatment and resuspended in PBS and serum-free DMEM, respectively. LLC cells (0.5 × 10^6^) were subcutaneously injected into the hind flank of 6-week-old C57Bl/6 wild-type and JDP2−/− mice. PyMT cells (2 × 10^6^) were orthotopically injected to the mammary fat pad as previously described [32]. Tumor size was assessed regularly with Vernier caliper using the formula width^2^ × length × 0.5. Unless otherwise specified, when tumors reached endpoints mice were sacrificed and tumors and lungs were harvested.

### Bone marrow transplantation

Bone narrow transplantation was performed as previously described [35]. Briefly, bone marrow cells were obtained by flushing femurs and tibias of 7–8 week old donor C57Bl/6 wild-type and JDP2−/− mice. The BMDCs (10 × 10^6^ per percipient) were transplanted by intravenous injection into lethally irradiated C57Bl/6 recipients. Irradiation was performed at 1000 cGy total body irradiation (250cGy/min) using Elekta Precise (ElektaOncology Systems) linear accelerator 6MeV photon beam radiation (Department of Radiation Therapy, Rambam Medical center, Haifa, Israel). After bone marrow cell reconstitution (usually 6–8 weeks), recipient mice were bled from the orbital sinus to evaluate bone marrow transplantation efficiency. Genomic DNA was extracted from white blood cells and PCR was performed with appropriate primers as previously described [19].

### Metastasis analysis

Lungs from tumor-bearing mice were harvested and embedded in paraffin. Lung sections were stained with Hematoxylin and Eosin (H&E) to evaluate metastatic lesions. The 3DHISTECH Pannoramic MIDI system (3DHISTECH Ltd.) was used to scan lung sections. Panoramic viewer was used to analyze metastatic lesions.

### Tumor immunostaining for hematopoietic cells

LLC tumors were obtained from wild-type and JDP2 −/− mice. Tumors were preserved in OCT (Sakure) and placed in −80°C. Tumor sections (8 μm thick) were prepared using Leica CM 1950 microtome (Leica). Tumor fixed tissues (4% paraformaldehyde) were stained for the pan-hematopoietic marker CD45 conjugated with R-phycoerythrin, and counterstained with DAPI (a nuclear marker). Images were acquired using a camera attached to an inverted microscope (Leica CTR6000) using Leica Application suite Version 3.4.0 software. The number of CD45^+^ cells was counted using Image J software.

### Conditioned medium

BMDCs were obtained by flushing femurs and tibias of 7–8 week old donor C57Bl/6 wild-type and JDP2−/− mice. The bone marrow cells were seeded in serum-free media at a concentration of 10 × 10^6^ cells/ml and cultured for 24 hours at 37°C in a humidified atmosphere containing 5% CO_2_. Medium was collected, centrifuged to remove cells, and frozen at −20°C for further use. For the preparation of a conditioned medium derived from cancer cells, LLC cells were seeded in serum-free media at a concentration of 10^6^ cells/ml and cultured for 24 hours at 37°C in a humidified atmosphere containing 5% CO_2_. Medium was collected and centrifuged to remove cells and cell debris. Samples were stored at −20°C for further use.

### Flow cytometry acquisition and analysis

Cell suspensions were assessed for T cells, macrophages, neutrophils and their subpopulations thereof using flow cytometry, as described previously [24]. Briefly, tumors removed from tumor-bearing mice were prepared as single-cell suspensions as previously described [35], and then immunostained with the following antibody mixture: CD3ε for T cells, F4/80 for macrophages, CD11b, Ly6G and Ly6C for neutrophils. Mature neutrophils were identified as CD11b^+^Ly6C^(high)^Ly6G^+^, and immature neutrophils as CD11b^+^Ly6C^(low)^Ly6G^+^. Flow cytometry experiments were performed using BD LSRFortessa™ (BD Bioscience) flow cytometer and analyzed with Summit version 4.3 (Beckman Coulter).

### Invasion and migration assays

Invasion and migration assays were performed using Boyden chambers as previously described [25]. Briefly, filters (6.5 mm in diameter, 8 μm pore size) were coated with either Matrigel (diluted 1:4 in DMEM, BD Bioscience, USA), or 0.01 μ /μl fibronectin (Biological Industries, Israel) for invasion or migration assay, respectively. Freshly coated filters were left to dry for 2 hours at 37°C. Serum-deprived LLC, PyMT cells were seeded in the upper compartment of the chamber and the lower compartment was filled with one of the following: serum-free medium, medium supplemented with 10% FBS, conditioned media collected from either wild-type or JDP−/− BMDC cultures. After incubation for 24 hours at 37°C, cells that passed through to the bottom filter were stained with 0.5% Crystal violet (Sigma-Aldrich) and photographed with an inverted microscope (Leica DMIL LED). Analysis of cell coverage was performed using Adobe Photoshop CS2 program. To test the effect of neutralizing CCL5 antibodies on invasion, 250 ng/ml anti-murine CCL5 antibody was added to conditioned media in the bottom chamber. cFOS rabbit polyclonal IgG was used as an IgG control. To test the effect of a combination of mouse CCL3, CCL4 and CCL5 (PeproTech Inc., Rocky Hill, NJ) on LLC invasion, 10 ng/ml and 100 ng/ml of each chemokine was added to JDP2−/− CM in the bottom chamber. To test the effect of addition of CCL5 alone on LLC invasion, 100 ng/ml of CCL5 was added to either JDP2−/− or wild-type CM.

### Isolation of bone marrow derived neutrophils

BM cells were obtained by flushing femurs and tibias of 7–8 week old donor C57Bl/6 wild-type and JDP2−/− mice. Neutrophils from BMDCs were obtained using a neutrophils isolation kit (Neutrophils Isolation Kit, Milteny Biotec Inc., Auburn, CA, USA). Pooled samples from 6 mice per group were used to obtain CM.

### Migration closure assay

PyMT cells (7 × 10^5^ cells/ml) were seeded onto ibidi Culture-Inserts (ibidi GmbH) and incubated at 37°C in a humidified atmosphere containing 5% CO_2_. After cell attachment, cells were placed in medium containing 5% serum overnight. Inserts were removed and medium was changed to the following treatments: DMEM supplemented with 10% serum, DMEM supplemented with 1% serum in the presence or absence of CM collected from either wild-type or JDP2−/− BMDC cultures. Cell migration towards the empty space left from the insert was monitored using Time-lapse system (Zeiss Hrm) with an on-stage incubator. Analysis was performed using Image-Pro Premier (Media Cybernatics).

### Cytokine array

Conditioned media collected from wild-type or JDP2−/− BMDC cultures were applied to a cytokine array (Proteome Profiler Mouse Cytokine Array Kit, panel A, R&D Systems Inc., Minneapolis, MN) according to the manufacturer's instructions. Detection was performed by ECL and cytokine levels quantified by densitometry.

### ELISA

Quantification of CCL5 protein secreted into CM was performed using a sandwich ELISA kit (DuoSet ELISA kit, R&D Systems Inc., Minneapolis, MN) according to the manufacturer's instructions. Quantification of CCL3 and CCL4 secreted into CM was performed with MINI ELISA kits (Murine MIP-1α/β Mini ELISA Development Kit, PeproTech, Inc., Rocky Hill, NJ) according to the manufacturer's instructions.

### MMP9 and MMP2 detection by gelatin zymography

Conditioned media (CM) collected as specified in the text and supplementary figure legends were analyzed for MMP9 and MMP2 activity using gelatin zymography. Equal amount of CM was loaded on 10% SDS-polyacrylamide gels containing 0.1 mg/ml gelatin and resolved at 125 volts at 4°C. The gel was then placed in re-naturation buffer (Bio-Rad) for 1 h and washed twice in DDW for 20 min each time. The gel was incubated overnight in development buffer (Bio-Rad) at 37°C, followed by soaking in 100 mM EDTA for 15 min, and fixation in 10% Acetic acid and 10% Methanol in DDW for 1 h. Finally, the gel was stained with Coomassie Blue solution and destained with a solution of 10% Acetic acid and 10% Methanol in DDW. Analysis was performed using Image lab 5.1 (Bio-Rad) and bands were quantified using TotalLabQuant (TotalLab Ltd). CM of HT-1080 cells were used as positive control for both MMP9 and MMP2.

### Protein extraction

LLC and PyMT cells were lysed in whole cell extract (WCE) buffer [25 mM HEPES pH 7.7, 0.3 M NaCl, 1.5 mM MgCl2, 0.2 mM EDTA, 0.1% Triton X-100, 0.5 mM DTT, 20 mM β-glycerophosphate, 0.1 mM Na2VO4, 100 μg/ml PMSF, Protease inhibitor cocktail 1:100 (Sigma Aldrich, P8340)].

### Western blot

Cell lysates were boiled in 95°C for 3 minutes. Lysates were separated by 10% SDS-PAGE, and electro-transferred to nitrocellulose membranes. Membranes were blocked in 5% dry milk in PBS for 0.5 hour, and then incubated with primary antibodies for αCCR5 (diluted 1:1000) and αGAPDH overnight at 4°C. After 3 washes, the membranes were incubated with HRP-conjugated anti rabbit secondary antibody for 1 hour at room temperature, followed by 3 washes. Protein bands were detected by enhanced chemiluminescence.

### Luciferase reporter assay

HEK-293T cells were co-transfected with the reporter plasmid pGL3, containing a portion of murine CCL5 promoter (from −1036 to +52), upstream of the luciferase gene (kindly provided by prof. Xian K. Zhang form the Medical University of South Carolina) [27]) and the pCEFL-HA-JDP2 expression vector. The total amount of plasmid DNA was adjusted to 12 μg. Cell culture medium was replaced with fresh medium 4–5 h post transfection. Cells were harvested 24 h post transfection. Subsequently, cell pellet was re-suspended in Promega lysis buffer. Cell lysate was tested for luciferase activity using the luciferase assay kit (Luciferase Reporter Assay System, Promega, CA, USA) and TD 20/20 luminometer (Turner Designs, Sunnyvale, CA, USA).

### Statistical analysis

Data are expressed as means ± SD, and the statistical significance of differences was assessed by one-way ANOVA, followed by Tukey post-hoc statistical test using GraphPad Prism 5 software (La Jolla, CA) or with unpaired student *t*-test, as indicated in the text. Differences between all groups were compared with each other or to control (in the case of student *t*-test), and were considered significant at values of *P < .05, **P < .01, and ***P < .001.

## SUPPLEMENTARY FIGURES


