# Supplementary material for: Host JDP2 expression in the bone marrow contributes to metastatic spread
Source: Oncotarget. 2015 Oct 14;6(35):37737–49. doi: 10.18632/oncotarget.5648 (PMC4741961; doi:10.18632/oncotarget.5648)
Supplement: Supplementary file 1 [file oncotarget-06-37737-s001.pdf]

SUPPLEMENTARY FIGURES

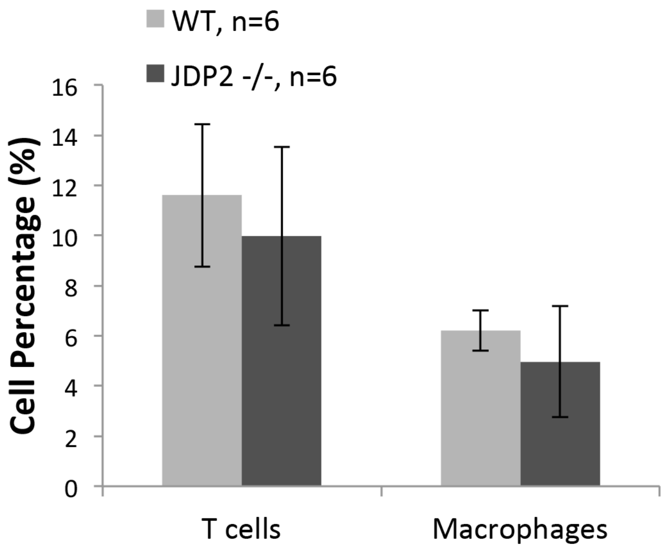

Supplementary Figure S1: Percentage of T cells and macrophages in tumors of wild-type and JDP2−/− mice. Six-to-eight week old wild-type and JDP2 −/− mice were subcutaneously implanted with 0.5 × 10^6 LLC cells. When tumors reached an average volume of 2000 mm^3, mice were sacrificed and tumors excised. Tumors were prepared as single cell suspensions and the percentage of T cells (CD3ε) and macrophages (F4/80) was determined by flow cytometry.

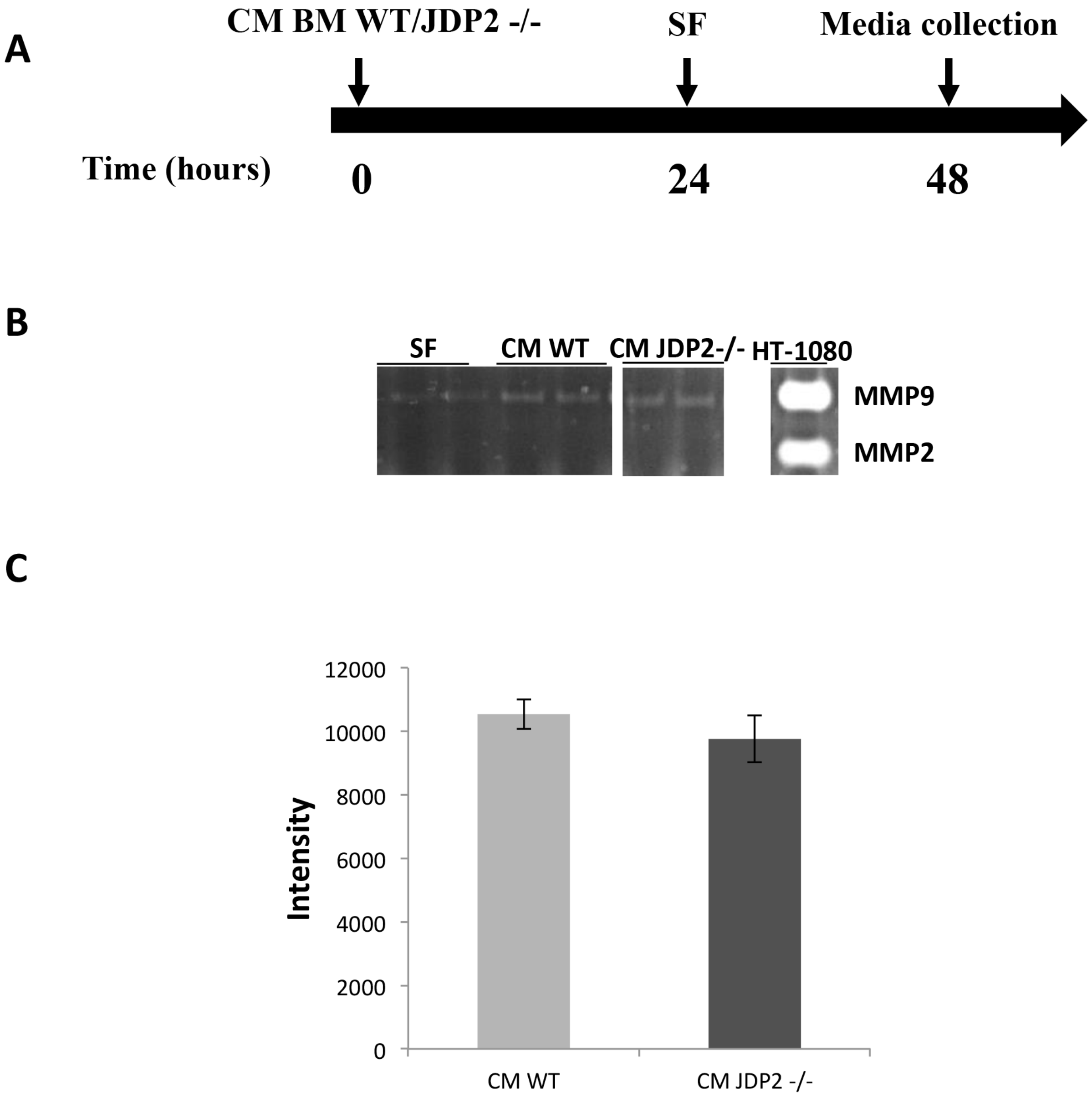

Supplementary Figure S2: MMP9 secretion from LLC cells exposed to conditioned medium of wild-type and JDP2−/− BMDCs. A. LLC cells were cultured in the presence of conditioned medium (CM) derived from wild-type or JDP2−/− BMDCs. After 24 h, the medium was replaced with serum-free (SF) medium, which was then collected 24 h afterwards. **B–C.** The activity of MMP9 in the CM was assessed by gelatin zymography. CM of HT-1080 was used as positive control (B), followed by densitometry (C)

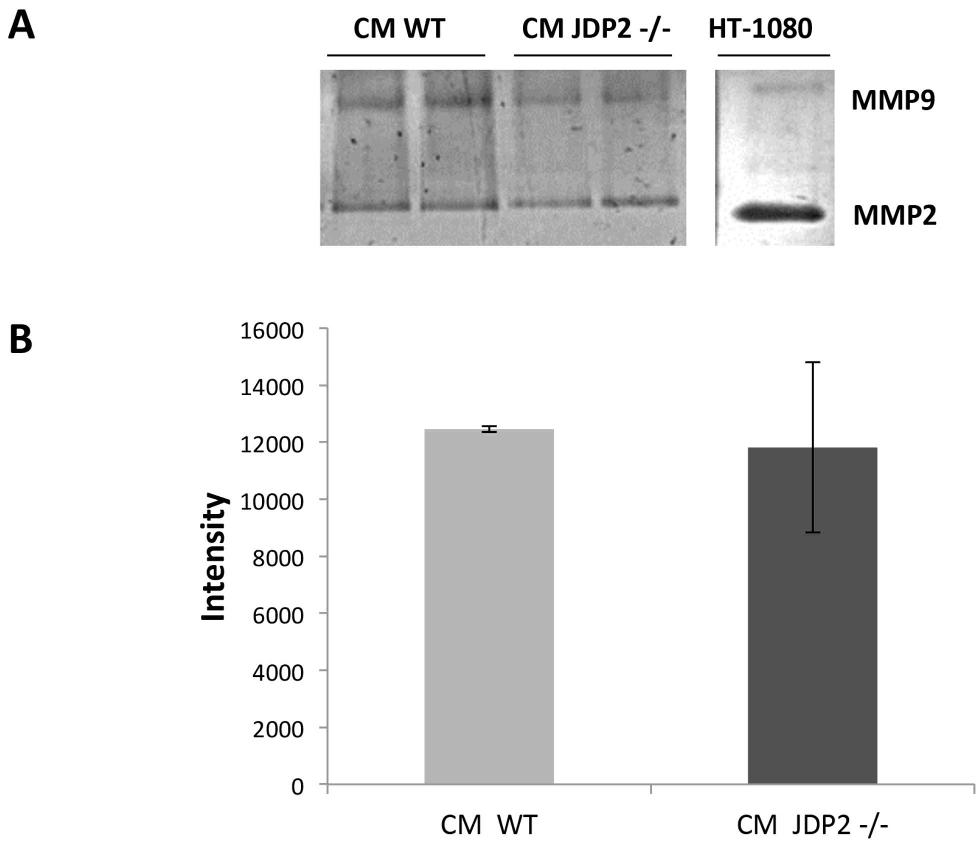

Supplementary Figure S3: MMP9 secretion from wild-type and JDP2−/− BMDCs. A–B. BMDCs derived from wild-type and JDP2−/− mice were cultured for 24 h and conditioned medium (CM) was collected. The activity of MMP9 in the CM was assessed by gelatin zymography. CM of HT-1080 was used as positive control (A), followed by densitometry (B)

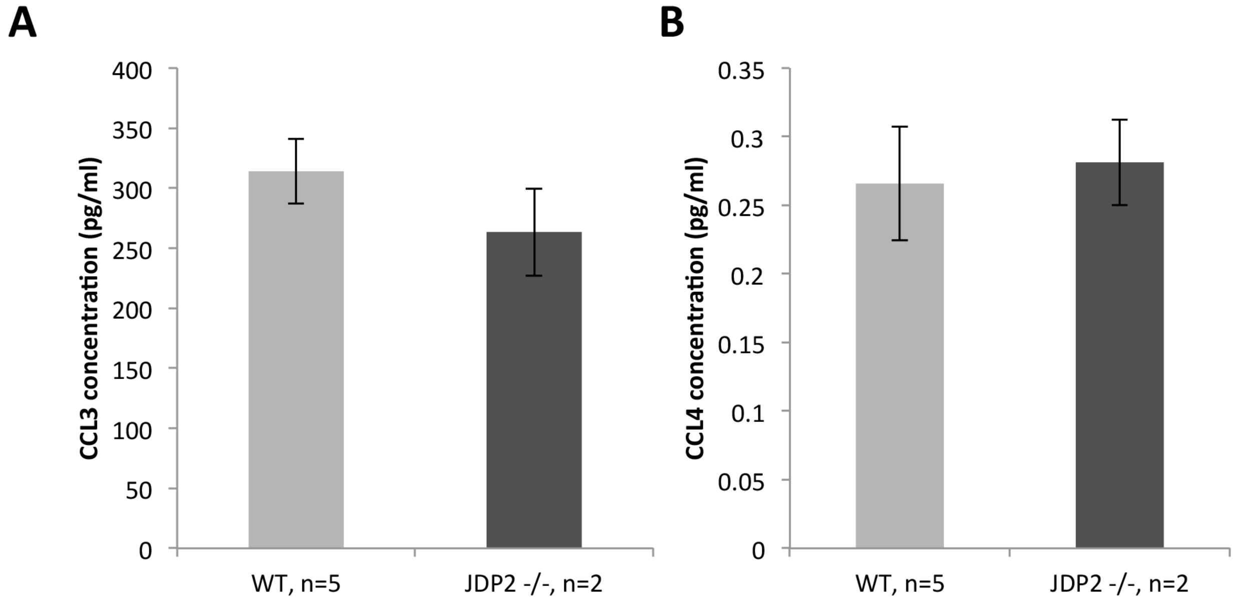

Supplementary Figure S4: CCL3 and CCL4 secretion from wild-type and JDP2−/− BMDC cultures. A–B. BMDCs derived from wild-type and JDP2−/− mice were cultured for 24 h and conditioned medium was collected. The level of secreted CCL3 (A) and CCL4 (B) in the conditioned medium was assessed by ELISA.

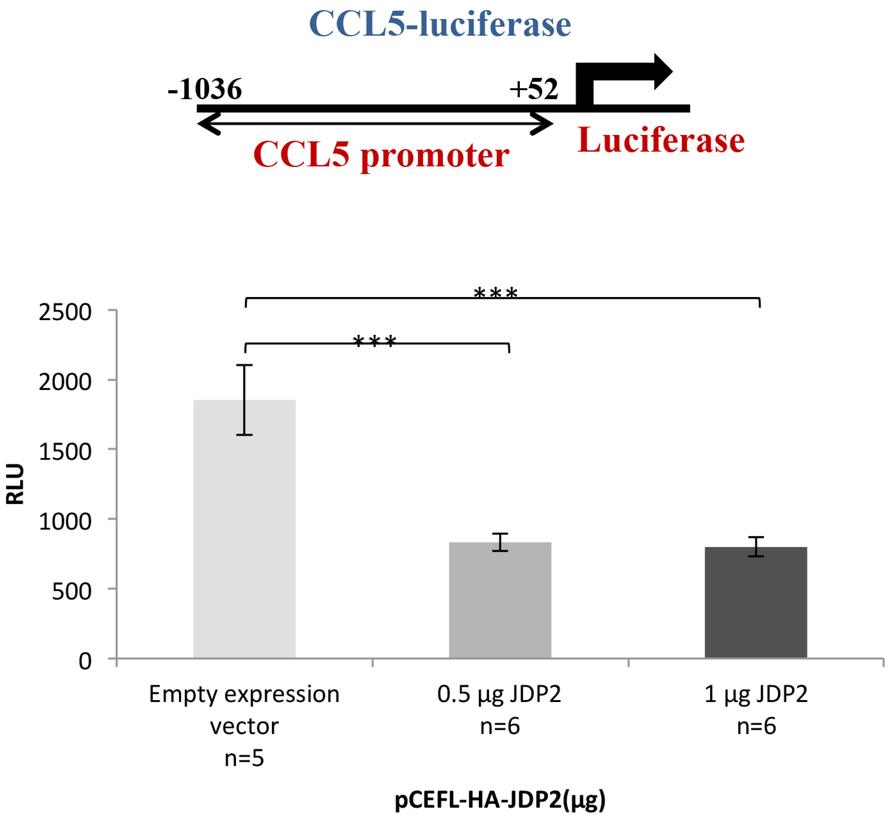

Supplementary Figure S5: JDP2 suppresses CCL5 transcription. HEK 293T cells were transfected with the CCL5-luciferase reporter plasmid in the presence or absence of pCEFL-HA-JDP2 expression plasmid. Luminescence was evaluated 24 h post transfection (B) Relative light unit (RLU) is indicated. ***, $p < 0.001$ using one way ANOVA followed by Tukey post-hoc test.

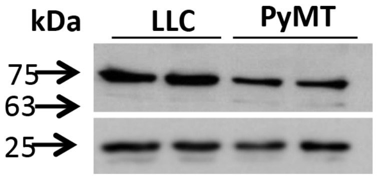

Supplementary Figure S6: CCR5 is expressed in cancer cells. Western blot analysis of cell lysate derived from LLC and PyMT cells probed with anti-CCR5 antibody. Anti-GAPDH serves as a loading control. The migration of the relevant proteins and molecular size markers are indicated by arrows.
